# Spatiotemporal Pattern and Convergence Test of Energy Eco-Efficiency in the Yellow River Basin

**DOI:** 10.3390/ijerph20031888

**Published:** 2023-01-19

**Authors:** Shan Feng, Yawen Kong, Shuguang Liu, Hongwei Zhou

**Affiliations:** 1 School of Economics, Ocean University of China, Qingdao 266100, China; 2 Institute of Ocean Development, Key Research Base of Humanities and Social Sciences, Ministry of Education, Qingdao 266100, China

**Keywords:** Yellow River Basin, energy eco-efficiency, Super-EBM, *σ*-convergence, *β*-convergence

## Abstract

Examining the convergence characteristics of energy eco-efficiency in the Yellow River Basin (YRB) is of great significance for the sustainable development of China. It fulfills the international commitment to carbon peak and carbon neutrality. Based on the Super-EBM model and ML index, this paper measures the energy eco-efficiency of 60 cities in the YRB during 2006–2018, and then spatial and temporal patterns are both analyzed before the final investigation of the convergence in the YRB. The results show the following: (1) From 2006 to 2018, the energy eco-efficiency of the YRB showed a significant upward trend, but there was still a 25.61% improvement compared with the production frontier. (2) The spatial differentiation of the energy eco-efficiency in the YRB was significant, and the inter-regional differences were the main reason for this. (3) There was no *σ*-convergence in energy eco-efficiency in the YRB during 2006–2018, but absolute and conditional *β*-convergence did occur. (4) Although the significant factors in the convergences were different, the levels of energy eco-efficiency in the different reaches all developed towards stable levels, and the catch-up effects in the less-developed regions were significant.

## 1. Introduction

Since the beginning of the Industrial Revolution, a clean, affordable, and reliable energy supply has been the cornerstone of the world’s growing prosperity and economic growth [1]. However, the world is currently experiencing significant changes that have not occurred in centuries. How to overcome the constraints brought about by global climate change and energy shortages has become the main concern in big-power politics. In particular, since February 2022, the energy crisis caused by the ongoing Russia–Ukraine conflict has swept Europe, placing the global energy market under attack. As a combination of energy efficiency and eco-efficiency [2], energy eco-efficiency is a measure of sustainability that refers to how well energy-consumption activity takes into account the ecological and economic benefits of a socioeconomic system, acting as an indicator that reflects the concept of the coordinated development of “energy–economy–environment–society” [3]. The promotion of energy eco-efficiency is seen as a major factor in reducing greenhouse gas emissions, as well as in maintaining the security of the energy supply.

As a major manufacturing country, China surpassed Japan and became the world’s second-largest economy in 2010. Support from energy was essential for this high-speed development. According to statistics, China’s primary energy consumption in 2020 was 145.46 EJ, which was 1.657 and 8.556 times that of the USA and Japan, respectively (BP Statistical Review of World Energy, 2021. https://www.bp.com.cn/content/dam/bp/country-sites/zh_cn/china/home/reports/statistical-review-of-world-energy/2021/BP_Stats_2021.pdf, accessed on 7 November 2022). In contrast to the high growth of its energy consumption, the energy-resource endowment of China is not high, and its per capita share is relatively low. Moreover, the traditional economic-orientated and high-pollution mode of development seriously hinders the sustainable development of China. Clearly aware of the potential problems, concepts of energy-intensity regulation, ecological civilization, and green economy have been proposed in China, and commitments to carbon peak and carbon neutrality by 2030 and 2060, respectively, have been made to the world. To achieve these goals, the locations in China where the conflicts between energy utilization and the ecological environment are fierce need attention.

The YRB is an important ecological barrier, economic area, and energy basin for China, and about 80% of China’s coal chemical enterprises are located in cities along the Yellow River, with 70% of coal mining taking place in the middle and lower reaches [4]. However, extensive utilization and excessive consumption pose a great challenge to the sustainable development of the basin’s economy, making it difficult to provide good support for Chinese modernization. Therefore, improving the energy eco-efficiency of the YRB is not only an objective requirement for achieving the goals of the ecological protection and high-quality development of the YRB and ensuring that “the rice bowl of energy must be in our own hands”, but also an inevitable choice to respond actively to global climate change and fulfill the international commitment to carbon peak and carbon neutrality.

## 2. Literature Review

The energy problem is a major concern for scholars all over the world. A large number of studies focus on the relationship between energy consumption and economic growth [5,6], national energy security evaluation, and path selection [7]. Meanwhile, as the key to breaking the constraints of energy shortages and environmental pollution, energy efficiency is also widely discussed [8]. Energy efficiency refers to a state of high output and low energy consumption [9]. Wu et al. (2014) [10], Filippini et al. (2016) [11], and Liddle et al. (2021) [12] measured and analyzed the energy efficiency of China, the United States, and economies inside and outside the Organisation for Economic Co-operation and Development (OECD) based on input–output data, such as capital, labor, energy, and GDP. Their conclusions provided the theoretical basis for this paper and the analysis of energy efficiency in different regions. However, the relevant studies prioritize economic benefits without comprehensively considering social welfare.

Energy eco-efficiency focuses on the ecological and environmental benefits offered by energy production, and the intensity of their environmental impact and resource utilization is required to match the carrying capacity of the Earth [13], which is better suited to the goals for sustainable development set by the United Nations (UN) and the construction of China’s ecological civilization. Studies on energy eco-efficiency are mainly based on two perspectives: The first focuses on the evaluation of the effectiveness of policy instruments. For example, Zhou et al. (2019) [14] stated that fiscal decentralization and economic competition among local governments can improve and decrease energy eco-efficiency, respectively. You et al. (2022) [15] found that under the constraints of environmental regulations, the industrial agglomeration had a single threshold effect on Chinese energy eco-efficiency. Song et al. (2022) [16] noted that innovation support provided by the government played an important role in promoting energy eco-efficiency by influencing regional industrial upgrading. The second perspective mainly discusses the current situation or the factors influencing energy eco-efficiency. For example, Yue et al. (2022) [17] concluded that with carbon emissions as the undesired output, energy eco-efficiency is much lower than energy efficiency. Tang et al. (2021) [18] proposed that technological progress is the major motivation for the growth of energy eco-efficiency in the Guanzhong Plain urban agglomeration. Guan et al. (2022) [19] confirmed that the economic growth and energy eco-efficiency in the YRB show a strong decoupling–weak decoupling–strong decoupling–weak decoupling development trend. In addition, industries with high energy consumption—such as mining [20], manufacturing [21], and logistics [22]—also raise concerns. Regarding research methods, parametric analysis based on stochastic frontier analysis (SFA) and non-parametric methods based on data envelopment analysis (DEA) are used to measure energy eco-efficiency. Moreover, the Tobit model, the geographical detector, and the spatial econometric model are used to explore the drivers of energy eco-efficiency. With the deepening research on energy eco-efficiency, its convergence has gradually become the most significant topic in the field of economics and the environment. For example, Meng et al. (2019) [23] concluded that there was spatial *β*-convergence in China’s energy eco-efficiency. Sheng et al. (2019) [24] noted that the energy eco-efficiency of urban agglomerations in the Yangtze River basin showed a downward trend, and the convergence in each region was different.

In summary, the existing literature holds promise for the further study of energy eco-efficiency and its trends in the cities in the YRB. However, the general convergence in cities of a particular basin remains challenging but highly desirable. The potential marginal contributions of this paper are as follows: (1) In terms of research objects, 60 cities above prefecture level in the YRB were selected, and the heterogeneity of energy eco-efficiency and convergence in the upper, middle, and lower reaches were analyzed. (2) In terms of the research methods, an input–output indicator system with undesirable outputs was constructed, and the spatiotemporal evolution characteristics and convergence of energy eco-efficiency in the YRB were comprehensively examined from the perspectives of geography and economics. (3) Theoretically, the main factors in and mechanisms behind the convergence of energy eco-efficiency were systematically analyzed. This work satisfies the urgent need for ensuring energy security and comprehensively promoting the ecological protection and high-quality development of the YRB.

## 3. Materials and Methods

### 3.1. Energy Eco-Efficiency Measurement

#### 3.1.1. Super-EBM

DEA is an effective tool to measure the efficiency of decision-making units (DMUs), with many classic forms, such as CCR (named after the initials of Charnes, Cooper, and Rhodes, 1978) [25], BCC (named after the initials of Banker, Charnes, and Cooper, 1984) [26], slacks-based measure (SBM) [27], etc. However, its single radial or non-radial assumption often differs from the reality, resulting in inefficiency factors. Therefore, Tone and Tsutsui (2010) [28] improved the model and proposed the EBM (epsilon-based measure) hybrid model, including radial- and non-radial distance functions, which has been widely used in the measurement of energy efficiency [29]. However, the problem of insufficient efficiency discrimination of DMUs on the frontier still exists. For this reason, this paper adopts the non-guided Super-EBM model with a comparable frontier DMU and undesirable outputs to evaluate the energy eco-efficiency of the YRB, as well as those of its upper, middle, and lower reaches. The method is shown in Equation (1).

(1) $$\begin{array}{l} E=\min\frac{\theta -\varepsilon_{x}\sum_{i=1}^{N}\frac{w_{i}^{-}s_{i}^{-}}{x_{ik}}}{\varphi +\varepsilon_{y}\sum_{r=1}^{q}\frac{w_{r}^{+}s_{r}^{+}}{y_{rk}}+\varepsilon_{b}\sum_{p=1}^{q}\frac{w_{p}^{b-}s_{p}^{b-}}{b_{pk}}}\\ s.t\left\{\begin{array}{l} \sum_{k=1}^{K}\lambda_{k}X+s_{i}^{-}=\theta x_{ik}\\ \sum_{k=1}^{K}\lambda_{k}Y-s_{r}^{+}=\varphi y_{rk}\\ \sum_{k=1}^{K}\lambda_{k}B+s_{p}^{b-}=\varphi b_{pk}\\ \lambda_{ik},s_{i}^{-},s_{r}^{+},s_{p}^{b-}\geq 0\\ i=1,2,\ldots ,N;\;r=1,2,\ldots ,M;\\ p=1,2,\ldots ,P; \end{array}\right. \end{array}$$

where E is the energy eco-efficiency value and λ is the weight vector. The x,y, and b denote the input, desired output, and undesired output, respectively. The θ denotes the radial efficiency value. The ε denotes the model parameter; if ε=0, the EBM model is equivalent to a radial model, and if ε=1, it is equivalent to a SBM model. The $s=\left(s_{i}^{-},s_{r}^{+},s_{p}^{-}\right)$ and $w=\left(w_{i}^{-},w_{r}^{+},w_{p}^{-}\right)$ represent the slack variables and relative weights of the input, expected output, and undesired output of the energy-utilization factors, respectively. For energy eco-efficiency, the DMU has not reached the valid state when 1>E≥0, while it is on the production frontier when E>1, and the higher the value of E, the higher the energy eco-efficiency.

#### 3.1.2. Malmquist–Luenberger Index

As a static method of evaluating the efficiency of cross-sections, it is impossible to reflect the dynamic changes of the same DUM using Super-EBM. Therefore, Fare et al. (1994) combined the Malmquist index with DEA, making dynamic efficiency evaluation possible [30]. Furthermore, Chung Y.H. et al. (1997) [31] introduced the directional distance function into the Malmquist index to deal with the undesirable output problems. This was named the Malmquist–Luenberger (*ML*) index. To fully explore the dynamic changes in energy eco-efficiency, the *ML* index was adopted in this study, and a further decomposition was conducted to investigate the reasons for changes, while ML=EC×TC, where EC denotes the efficiency change and TC denotes the technology change. When the ML index is greater than 1, this indicates an increase in energy eco-efficiency from period t to period t+1; otherwise, it means a decrease. The specific calculation formula is provided in a previous study [32].

### 3.2. Spatial Difference Analysis

#### 3.2.1. Gravity Standard Deviational Ellipse Analysis

With the standard deviational ellipse (SDE) method proposed by Lefever [33], four basic parameters—namely, barycentric coordinates $\left(G\left(x,y\right)\right)$, rotation angle (tanθ), and the standard deviation of the *x*-axis ($\sigma_{x}$) and *y*-axis ($\sigma_{y}$)—were used to explore the spatial distribution characteristics of energy eco-efficiency in the YRB, such as the main spatial location, development trend, and dispersion degree in the main and secondary directions. Specific equations are shown in Table 1.

#### 3.2.2. Dagum’s Gini Coefficient Analysis

In this study, Dagum’s [34] method was used to measure and decompose the regional differences in energy eco-efficiency in the YRB. According to the differences between the measured subjects, the calculation can be divided into the overall Gini coefficient (G), the overall regional difference Gini coefficient ($G_{jj}$), and the regional difference Gini coefficient ($G_{jh}$). The G can be decomposed into the intra-regional gap ($G_{w}$), inter-regional gap ($G_{nb}$), and super-variable density contribution ($G_{t}$), which fulfill $G=G_{w}+G_{nb}+G_{t}$. Specific equations are shown in Table 2.

### 3.3. Convergence Calculating

#### 3.3.1. *σ*-Convergence Model

The σ-convergence reflects the dispersion degree of different groups. This study used the coefficient of variation to test whether the deviation of energy eco-efficiency in the YRB tended to shrink over time. The method is shown in Equation (2).

(2) $$\sigma_{}=\frac{\sqrt{\left[{\sum_{i=1}^{n}\left(E_{i}-\bar{E_{}}\right)}^{2}\right]/n}}{\bar{E}}$$

where $E_{i}$ and $\bar{E}$ denote the energy eco-efficiency of city i and its mean value, respectively, while n represents the number of cities in the basin. If the coefficient of variation decreases with the passage of time, this indicates that the energy eco-efficiency of the YRB has σ-convergence characteristics; otherwise, it does not.

#### 3.3.2. *β*-Convergence Model

The *β*-convergence model can be divided into absolute *β*-convergence and conditional *β*-convergence. Absolute *β*-convergence assumes that different cities have the same development conditions, and cities with lower energy eco-efficiency have higher growth rates than cities with higher energy eco-efficiency, so that all cities converge. Conditional *β*-convergence takes the condition differences between regions into account while analyzing whether the energy eco-efficiency in the basin converges to its own stable level over time. The method is shown in Equation (3).

(3) $$\ln\left(\frac{E_{i,t+1}}{E_{i,t}}\right)=\alpha +\beta \ln E_{i,t}+\sum_{k=1}^{n}\theta_{k}X_{k,i,t}+\varepsilon_{i,t}$$

where α is the intercept term and β is the judgment coefficient term of convergence. If β<0, convergence of energy eco-efficiency exists, meaning that cities with lower energy eco-efficiency tend to catch up with cities with higher energy eco-efficiency; otherwise, there is no *β*-convergence. The $E_{i,t}$ and $E_{i,t+1}$ denote the energy eco-efficiency of city i in year t and year t + 1, respectively. The $\theta_{k}$ is the estimated coefficient of the *k*th control variable $X_{k,i,t}$. When $\theta_{k}=0$, it is an absolute *β*-convergence model; otherwise, it is a conditional *β*-convergence model. The $\varepsilon_{i,t}$ denotes the stochastic error term. The convergence rate is $-\ln\left(1+\beta \right)/T$, while T is the convergence time length.

### 3.4. Research Area and Data Source

#### 3.4.1. Research Area

As the second-longest river in China, the Yellow River flows from west to east through nine provinces (autonomous regions) and winds its way into the Bohai Sea. With reference to the division from the natural basin scope of the Yellow River by the Yellow River Water Resources Committee of the Ministry of Water Resources, considering that Sichuan was incorporated into the national strategy of the Yangtze River Economic Belt, along with the objective fact that the cities of Haidong and Laiwu adjusted their administrative divisions during the study period, drawing on relevant studies [35,36,37], 60 prefecture-level cities in eight provinces (autonomous regions)—namely, Qinghai, Ningxia, Inner Mongolia, Shanxi, Shaanxi, Henan, and Shandong—were finally selected as the research objects. These cities were divided into upper, middle, and lower reaches, with the town of Hekou in Inner Mongolia and the Taohua Valley in Henan as the demarcations (Upper reaches include Xining, Lanzhou, Baiyin, Wuwei, Dingxi, Longnan, Yinchuan, Shizuishan, Wuzhong, Guyuan, Zhongwei, Huhhot, Baotou, Ordos, Bayannur, Ulanqab, and Wuhai. Middle reaches include Tianshui, Pingliang, Qingyang, Xi’an, Xianyang, Tongchuan, Weinan, Yan’an, Yulin, Shangluo, Baoji, Taiyuan, Datong, Shuozhou, Xinzhou, Yangquan, Lvliang, Jinzhong, Changzhi, Jincheng, Linfen, Yuncheng, Sanmenxia, Luoyang, and Jiaozuo. Lower reaches include Zhengzhou, Kaifeng, Anyang, Hebi, Xinxiang, Puyang, Shangqiu, Jinan, Qingdao, Zibo, Weifang, Dongying, Jining, Taian, Dezhou, Liaocheng, Binzhou, and Heze).

#### 3.4.2. Data Source

(1) Input–Output Indicator System

Energy eco-efficiency reflects the effective utilization of factors in links between economic output and energy input under environmental constraints. Under certain conditions, the fewer the undesirable outputs, the higher the energy eco-efficiency. On the basis of clarifying the above concepts, as well as the research method of regional economic geography [38] and the overall representation and availability of data, the input–output indicator system of energy eco-efficiency in the YRB was constructed. This is shown in Table 3.

Input: Capital and labor are always the basic elements of the production function [39]. Considering the objective reality that the proportion of energy consumption in the Chinese manufacturing industry has always been high [40], indices of capital stock of fixed assets and the number of employees in secondary industry were selected. Energy input was represented by comprehensive energy consumption, so as to eliminate the inconsistency in types and dimensions.

Output: Both desirable economic growth and undesirable environmental damage were considered. Desirable output was represented by secondary GDP, and industrial SO_2_, industrial soot, industrial wastewater, and CO_2_ emissions were used to examine the impact on the environment.

(2) Environmental Variables

To study the conditional convergence of energy eco-efficiency, it is necessary to consider the influence of natural endowment, as well as economic and social development. Since the influence of economics, labor, and energy on the input–output indicator system did not need to be verified again, with reference to the existing research [41,42,43], and taking the availability of data into consideration, the influences of the following five aspects on the convergence of energy eco-efficiency in the YRB were examined:

Industrial Structure (STR): Upgrading industrial structure is an important way to achieve coordinated development between the economy and environment, promoting energy eco-efficiency. Therefore, the ratio of tertiary industry to secondary industry is used to represent it.

Influence of Government (GOV): By means of policy and financial support, the government can induce enterprises to gather, which further affects the energy eco-efficiency. Therefore, the ratio of fiscal expenditure to GDP in the year-end budget is used to represent it.

Opening Up (OPE): An open-circulation environment helps to establish direct links between international factors and domestic industries and technologies, helping to improve energy eco-efficiency. Therefore, the proportion of actual utilized foreign capital in GDP is used to represent it.

Urbanization (URB): Cities are the core energy-consuming regions. Improving urbanization can not only boost energy consumption objectively, but also improve energy eco-efficiency through scale economy. Therefore, the ratio of urban population to total population at the end of the year is used to represent it.

Innovation (INN): Improvements in technical levels bring about progress in equipment efficiency, which directly affects the improvement in energy eco-efficiency [44]. Therefore, the number of patent applications accepted is used to represent it.

(3) Data Source

The capital stock of fixed assets was measured by referring to the sustainable inventory method of Zhang et al. [45]. The comprehensive industrial energy consumption was converted by referring to the General Principles for the Calculation of Comprehensive Energy Consumption (GB/T 2589-2020), and the coefficients for gas, liquefied petroleum gas, and electricity consumption were 1.330, 1.714, and 0.123, respectively. The numbers of invention patents, utility model patents, and design patents were obtained through the CNRDS database and then converted by using the method of Bai et al. [46]. Weights of 0.5, 0.3, and 0.2 were assigned, respectively, so as to overcome the inconsistency in the degree of innovation. Moreover, pair numbers were taken to reduce heteroscedasticity problems. Spatial data were derived from prefecture-level vector maps. Price variables were all reduced based on 2006, and some missing data were supplemented by linear interpolation methods. Considering that the construction of a resource-saving and environmentally friendly society was set as a strategic task at the Fifth Plenary Session of the 16th CPC Central Committee in 2005, 2006 was chosen as the starting point of the study, and the sample investigation period of this paper was determined as 2006–2018.

## 4. Results

### 4.1. Spatiotemporal Pattern Analysis of Energy Eco-Efficiency

#### 4.1.1. Energy Eco-Efficiency Measurement and Evolution over Time

**Figure 1:**
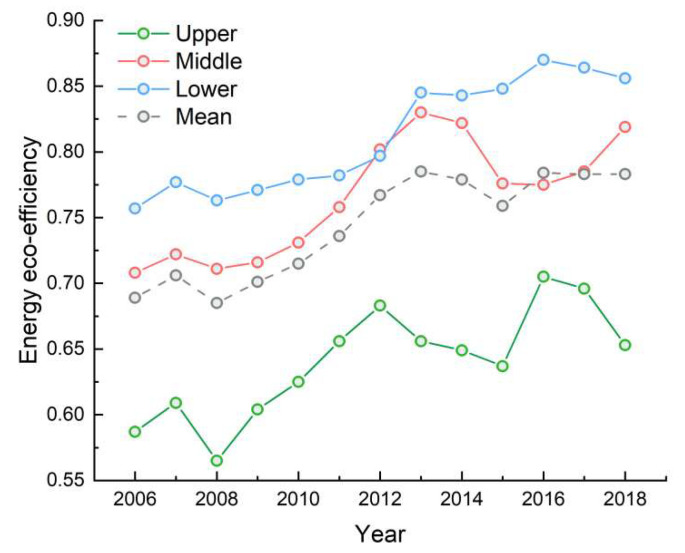
Energy eco-efficiency in the YRB from 2006 to 2018.

Based on the Super-EBM model, using Max DEA 8.0 software, the energy eco-efficiency in the YRB during 2006–2018 was measured, as shown in Figure 1. Overall, the energy eco-efficiency in the YRB showed an upward trend of fluctuation, with the average value increasing from 0.689 to 0.783 during 2006–2018. Specifically, after a steady increase from 2006 to 2013, the energy eco-efficiency fell back in 2014–2015, and then rose again from 2016 to 2018.

The ranking of energy eco-efficiency in the different reaches, from highest to lowest, was lower reaches, middle reaches, and upper reaches, with mean values of 0.811, 0.767, and 0.638, respectively, during 2006–2018. In particular, the energy eco-efficiency performed at ideal levels in the lower reaches and fluctuated around the mean value in the middle reaches, where the maximum value of 0.830 was attained in 2013. However, the energy eco-efficiency in the upper reaches was always lower than the mean level and fluctuated greatly.

Furthermore, the cities with effective energy eco-efficiency during the study period were Qingyang, Dongying, Ordos, and Yulin, which are resource-based cities, while the energy eco-efficiency in Longnan, Guyuan, and Dingxi was relatively low, with average values of less than 0.500.

The ML index was further used to conduct a dynamic analysis of the energy eco-efficiency in the YRB. The results are shown in Table 4. Overall, the mean value of the ML index in the YRB from 2006–2018 was 1.076, indicating that the development trend of energy eco-efficiency in the YRB was moving well. It was obvious that the ML index from 2011 to 2015 was less than 1. The reason for this may lie in the superimposed impact of the international financial crisis and industrial structure transformation, leading to slow economic growth and low energy eco-efficiency [47].

The ML index decomposition presented a dual-driven growth model of EC and TC, with growth rates of 3.3% and 4.1%, respectively. However, it was also noticeable that the performance was inconsistent across the reaches. The EC had a stronger driving effect than the TC in the upper and middle reaches, while in the lower reaches they advanced synchronously. These results were consistent with the basic background, according to which the rich energy resources in the middle reaches led to high resource-dependence and the extensive development of traditional modes, while the lower reaches were rich in innovative resources. From the perspective of the cities, the rapid improvement of TC and EC during the study period caused the energy eco-efficiency of Yulin, Zhongwei, Yan’an, Xi’an, Xianyang, Heze, Pingliang, and Guyuan to improve significantly, supporting an increase of more than 10%. However, cities such as Jining, Binzhou, Weifang, and Xining improved their energy eco-efficiency by less than 1%, and Tongchuan did not achieve any energy eco-efficiency improvements.

#### 4.1.2. Spatial Pattern Evolution of Energy Eco-Efficiency

**Figure 2:**
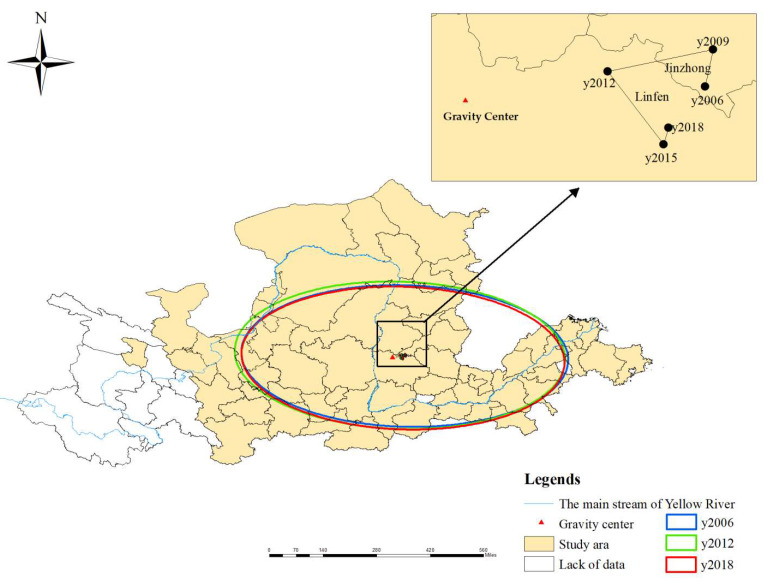
Shifting routes of energy eco-efficiency gravity centers and SDEs in the YRB.

The visualization of the shifting routes of the energy eco-efficiency’s gravity centers and SDEs can help to explore the spatiotemporal distribution patterns in the YRB. Using ArcGIS 10.2 spatial analysis technology, the results were calculated, as shown in Table 5 and Figure 2.

The gravity centers of energy eco-efficiency in the YRB were between 111.443° E~111.682° E and 36.593° N~36.773° N, and they were always eastward of the geometric gravity center; the average distance was 35.56 km. The shifting routes of the gravity centers showed a small range of zigzagging fluctuation of the C type. They occurred in Jinzhong from 2006 to 2009 and in Linfen for the remaining years, which showed movement in the southwest–southeast direction, with a total distance of 73.145 km. This indicates that the energy eco-efficiency improvements in the middle and lower reaches of the YRB were significantly higher than that in the upper reaches.

As shown in Table 5, the direction angle increased from 91.6358° to 92.5379° during 2006–2009, after which it decreased to 91.9337° in 2018, with an overall performance of 0.2979° clockwise deflection from southeast to due south. The standard deviation of the *x*-axis was greater than that of the *y*-axis, and it shortened after the initial extension, while the change on the *y*-axis was relatively small. These changes indicated that the spatial pattern of the energy eco-efficiency in the YRB was relatively stable in the north–south direction. Regarding the west–east main axis, a diffusion–assemble state was obvious. As a result, the ellipse area increased at the beginning and then decreased, which contributed to the increase–decrease changes in the overall spatial gap of the energy eco-efficiency, although the gap was still larger than that of the initial level.

To further reveal the temporal and spatial differences, as well as the sources of energy eco-efficiency in the YRB, Dagum’s Gini coefficient and its decomposition were used. The results are shown in Table 6.

In general, the overall Dagum’s Gini coefficient of the YRB’s energy eco-efficiency fluctuated between 0.106 and 0.146, and it showed a continuous decline from 0.115 to 0.106 during 2006–2012. Subsequently, it showed an upward trend, reaching a peak of 0.147 in 2017. It can be seen that the overall regional differences in the YRB showed U-shaped fluctuation within the study period, and the imbalance in the energy eco-efficiency in the YRB was continuously strengthened.

From the perspective of intra-regional differences, taking 2011 as the boundary, before that, the differences in the lower reaches were the highest, while the difference between the middle and upper reaches fluctuated around 0.1, showing a low level of inter-regional difference. This indicates that, compared with other regions, the differences within the lower reaches were greater. Subsequently, the Gini coefficient in the middle reaches exceeded that of the lower reaches, which meant that the difference within the middle reaches intensified. Meanwhile, the Gini coefficient in the upper reaches was relatively small, indicating that the equilibrium there changed little.

The level of inter-regional difference tended to intensify within the study period, corroborating the overall performance of the Gini coefficient. Compared with 2006, the inter-regional Gini coefficient of the upper–middle reaches increased from 0.107 to 0.145 in 2018—a growth of 35.514%—indicating that the difference in energy eco-efficiency between the middle and upper reaches increased significantly. Meanwhile, the change was relatively small between the middle and lower reaches, increasing from 0.123 to 0.138—a growth of 12.129%.

From the perspective of contribution rates, the intra-regional differences were relatively stable, fluctuating at around 32%, but the super-variable densities were the highest during the study period. This indicates that, in terms of energy eco-efficiency, there was a clear dividing line between the upper, middle, and lower reaches. The average contribution rates of the intra-regional, inter-regional, and super-variable density were 32.47%, 26.93%, and 40.60%, respectively. It can be concluded that inter-regional differences are the main sources of overall differences in energy eco-efficiency in the YRB.

### 4.2. Convergence Test of Regional Differences

#### 4.2.1. *σ*-Convergence Analysis

**Figure 3:**
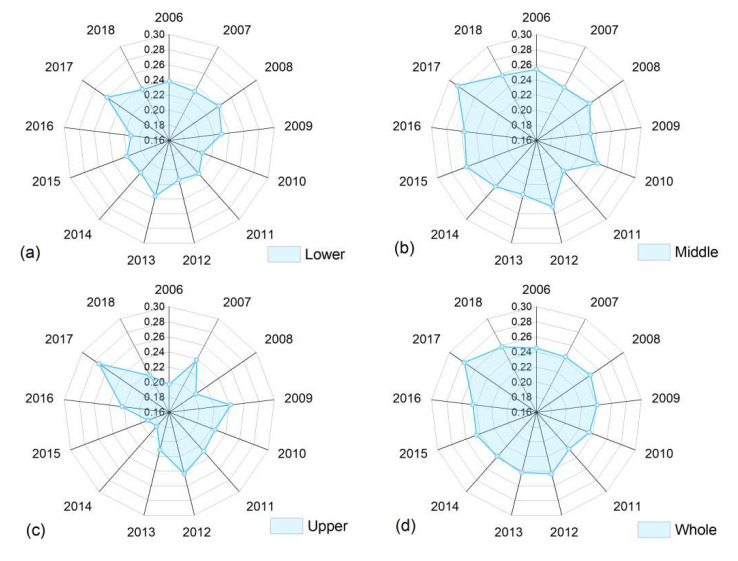
The *σ*-convergence test results for the YRB during the period from 2006 to 2018; (**a**–**d**) are *σ*-convergence test results for the lower, middle, and upper reaches and the whole basin, respectively.

In accordance with Equation (2), Figure 3 shows the *σ*-convergence results of 60 cities from 2006 to 2018. From the perspective of the evolution trend, the variation coefficients of the energy eco-efficiency in the YRB generally fluctuated in a declining–rising–declining fashion over time. The energy eco-efficiency increased from 0.245 in 2006 to 0.258 in 2018, meaning that there was no significant *σ*-convergence overall. Specifically, the increases in the variation coefficients in the upper, middle, and lower reaches were 9.224%, 1.070%, and −0.436%, respectively, indicating that the *σ*-convergence only existed in the lower reaches, with a weak intensity. Meanwhile, the variation coefficients in the middle reaches were relatively large, which suggests that the energy-utilization levels of the cities in the middle reaches varied greatly. These results are generally consistent with the above conclusions that the regional imbalance in energy eco-efficiency in the YRB increased after 2011 and that the Gini coefficient in the middle reaches was higher.

#### 4.2.2. Absolute β-Convergence Analysis

Using Stata 16 software, we tested the absolute β-convergence of energy eco-efficiency in the YRB using Equation (3), with $\theta_{k}=0$. Table 7 presents the results.

The absolute β-coefficients were significantly negative at the level of 1%, indicating that there was absolute β-convergence in the energy eco-efficiency in the YRB. This means that under the same conditions—such as industrial structure, government influence, openness to the outside world, urbanization level, and innovation level—the energy eco-efficiency of each city would eventually converge to the same steady-state level over time; that is, compared with the cities that enjoy higher energy eco-efficiency, the lower cities experienced a “catch-up effect”. These conclusions also apply to the upper, middle, and lower reaches. The convergence rates in the upper, middle, and lower reaches were 0.051, 0.026, and 0.040, respectively. It is clear that the upper reaches were the fastest, followed by the lower and middle reaches. However, these conclusions are based on the assumption that the same external factors applied, which is not consistent with the reality. Therefore, a conditional β-convergence test was needed for further verification.

#### 4.2.3. Conditional β-Convergence Analysis

Using Stata 16 software, we tested the absolute β-convergence of the energy eco-efficiency in the YRB using Equation (3), with $\theta_{k}\neq 0$. The results in Table 8 can be summarized as follows:

When considering the five control variables, the values of energy eco-efficiency in the YRB, as well as in the upper, middle, and lower reaches, were all negative and passed the significance test by more than 5%, further indicating the existence of β-convergence in the energy eco-efficiency in the YRB. Regarding the convergence rates, the upper, middle, and lower reaches displayed values of 0.078, 0.033, and 0.056, respectively; the upper reaches were still the fastest. Compared with the absolute β-convergence results, both the convergence rate and the goodness of fit improved. This is because the heterogeneity between the regions was considered in the β-conditional convergence model, which shortened the cycle of convergence, making the convergence test of the energy eco-efficiency more accurate and accelerating the convergence speed.

Specifically, the control variables in the YRB, as well as in the upper, middle and lower reaches, showed significant regional heterogeneity.

(1) The regression coefficients of the URB in the YRB and upper reaches were positive and significant at the 10% level, showing that the increase in the urbanization level increased the energy eco-efficiency but prevented the intra-regional gap from narrowing. Meanwhile, its influence on the middle and lower reaches was difficult to judge. A possible reason for this is that, for the upper reaches, the increase in urbanization level was often accompanied by improvements in infrastructure. In particular, the agglomeration of human flow, logistics, and capital flow with the support of the new round of the Western development policy was conducive to the achievement of scale benefit and energy eco-efficiency [48]. However, the middle and lower reaches have already achieved a relatively high level of urbanization by virtue of their location and resource advantages, and they are developing into a new type of urbanization, integrating urban and rural features. Therefore, the effects of “new urbanization” on the energy eco-efficiency in the YRB—based on the complementarity and coordination between speed and quality, economy and society, and urban and rural areas—deserve more attention.
(2) The regression coefficients of the $LN\left(INN\right)$ in the YRB and lower reaches were positive and significant at the 5% level, meaning that the increase in innovation levels increased the energy eco-efficiency but prevented the intra-regional gap from narrowing. Meanwhile, its influence on the middle and upper reaches was difficult to judge. The reasons for this may lie in the extensive use of the traditional economy and the absence of overall planning in the upper and middle reaches, resulting in seriously disordered development [49]; the levels of science and technology create significant bottlenecks in these areas. In contrast, the lower reaches’ advantage in terms of human capital was prominent. For example, Taishan Scholar, the Taishan Industry Leading Talents Project, and the Expert Workstation of the “Thousand Talents Plan” were launched in Shandong Province. These projects strongly supported technical innovation, improving the region’s energy eco-efficiency while optimizing the inputs of capital and labor.
(3) The regression coefficients of the OPE in the YRB and its upper and middle reaches were negative and significant at the 10% level, indicating that the increase in opening up promoted the convergence of energy eco-efficiency. Meanwhile, its influence on the lower reaches was difficult to judge. An open-circulation environment helps to establish direct links between international factors and domestic industries and technologies. A reasonable explanation for the this phenomenon is that, for the upper and middle reaches, the advanced technology and management experience brought by foreign investments can reduce the differences in local energy eco-efficiency through technology diffusion and knowledge spillover. For the lower reaches, on the one hand, their requirements from foreign investors are strict; on the other hand, given that their technological development is at a relatively advanced level, foreign investments that are based on trade protectionism have no significant positive effects on the convergence of energy eco-efficiency.
(4) The regression coefficients of the GOV in the YRB and its upper, middle, and lower reaches were not significant, indicating that the effects of government influence on energy eco-efficiency and regional differences cannot be clearly judged, and that further research is needed. The reason for this is probably that the surface industrial agglomeration is mainly formed by local governments’ inducement policies, such as financing, land, and tax allowances, which do not follow the market law. Due to this “free-rider” tendency, environmental governance and capital-reflected technological progress cannot exert their expected effects in improving the energy eco-efficiency [50].
(5) The regression coefficients of the STR in the YRB and its upper and middle reaches were negative and significant at the 1%, 1%, and 10% levels, respectively, showing that the upgrading of the industrial structure encourages the convergence of energy eco-efficiency. Meanwhile, its influence on the lower reaches was difficult to judge. As the “energy basin” of China, the YRB enjoys an outstanding resource endowment. The middle reaches, in particular, have long been agglomeration areas for coal, steel, and other heavily polluting industries. The upgrading of the industrial structure—against the background of Western development, supply-side structural reform, energy revolution, and commitments to carbon peak and carbon neutrality—is helpful in improving the energy eco-efficiency. However, in the lower reaches—especially in Jinan, Qingdao, and Zhengzhou—the service-oriented tertiary industry has a strong driving effect on the local economy, resulting in a subtle convergence effect of the industrial structure on the energy eco-efficiency.

## 5. Discussion

### 5.1. Revisiting Energy Eco-Efficiency and Convergence of Cities in the YRB

Improving energy eco-efficiency is of great significance to China’s high-quality economic development. A number of studies discuss the spatiotemporal evolution and influencing factors from national, provincial, industrial, and urban perspectives [51,52,53,54]. Some studies consider the existence of undesirable outputs and introduce them into an envelope analysis framework, which is suitable for measuring energy eco-efficiency. As a special economic system, the framework of energy eco-efficiency and convergence in the cities in a specific basin remains challenging. Thus, in this study, we relaxed the applicability and built an input–output indicator system to measure energy eco-efficiency. Furthermore, 60 cities in the YRB were selected as research areas to measure their energy eco-efficiency and driving forces during 2006–2018. The differences in the spatial patterns and evolution of the cities were analyzed, and the convergence was explored using *σ*-convergence analysis, absolute. *β*-convergence analysis, and conditional *β*-convergence analysis. This work improves the applicability of the energy eco-efficiency and convergence framework and enriches the existing body of research. The results can provide theoretical support for decision-makers.

### 5.2. Limitations and Potential Solutions

In this paper, we measured the energy eco-efficiency and driving forces in the YRB based on the Super-EBM model and ML index, analyzed the spatial evolution law of energy eco-efficiency by combining economics and geography, and used the coefficient of variation and panel convergence model to test the convergence characteristics. However, due to the lack of official data in some cities, this study only covered the most important energy types, resulting in low overall energy consumption. We intend to improve the integrated energy consumption accounting model, use night-light data, and use the latest official datasets in future studies. In addition, this paper focuses on the energy eco-efficiency of the YRB and its reaches; comparative studies on different industries could be added in the future.

## 6. Conclusions

By constructing an input–output indicator system involving undesirable outputs, this paper presents an empirical study on the convergence of energy eco-efficiency using a large-dimension spatial panel data model based on data from the period of 2006–2018 in the YRB. This work can provide guidance for the high-quality development of cities in the YRB. The four main conclusions drawn from this paper are as follows: 

First, from the perspective of temporal evolution, the energy eco-efficiency in the YRB showed a fluctuating increase from 2006 to 2018, but there was still a 25.61% improvement compared with the production frontier. The TC and EC jointly drove the improvement in energy eco-efficiency in the YRB, while the dominant factors in different reaches were not the same.

Second, from the perspective of the spatial pattern evolution, the imbalance in energy eco-efficiency in the YRB reflects the heterogeneity of the region. Compared with the middle and lower reaches, the energy eco-efficiency in the upper reaches showed a stronger balance, with a lower level. Meanwhile, the gravity center of the energy eco-efficiency in the YRB presented a small range of zigzag fluctuation of the C type.

Third, from the perspective of the convergence characteristics, there was no σ-convergence in energy eco-efficiency in the YRB during 2006–2018, but absolute and conditional β-convergence were observed. This means that there was a “catch-up effect” among the cities, and that the energy eco-efficiency in the YRB will trend towards a steady-state level over time. The convergence rate of the upper reaches was the highest, followed by the lower and middle reaches.

Fourth, regarding the results of the conditional β-convergence, the influences of the five factors were different. In general, openness and industrial structure significantly increased the convergence of the energy eco-efficiency in the YRB, while the levels of innovation and urbanization limited it.

## Figures and Tables

**Table 1:** Gravity standard deviational ellipse equations.

Index	Formulas
$G\left(x,y\right)$	$\left[\frac{\sum_{i=1}^{n}\omega_{i}\times x_{i}}{\sum_{i=1}^{n}\omega_{i}},\frac{\sum_{i=1}^{n}\omega_{i}\times y_{i}}{\sum_{i=1}^{n}\omega_{i}}\right]$
tanθ	$\frac{\left(\sum_{i=1}^{n}\omega_{i}^{2}{\tilde{x}}_{i}{}^{2}-\sum_{i=1}^{n}\omega_{i}^{2}{\tilde{y}}_{i}{}^{2}\right)+\sqrt{{\left(\sum_{i=1}^{n}\omega_{i}^{2}{\tilde{x}}_{i}^{2}-\sum_{i=1}^{n}\omega_{i}^{2}{\tilde{y}}_{i}{}^{2}\right)}^{2}+4\sum_{i=1}^{n}\omega_{i}^{2}{\tilde{x}}_{i}{}^{2}{\tilde{y}}_{i}{}^{2}}}{2\sum_{i=1}^{n}\omega_{i}^{2}{\tilde{x}}_{i}{\tilde{y}}_{i}}$
$\sigma_{x}$	$\sqrt{\frac{\sum_{i=1}^{n}{\left(\omega_{i}{\tilde{x}}_{i}\cos\theta -\omega_{i}{\tilde{y}}_{i}\sin\theta \right)}^{2}}{\sum_{i=1}^{n}\omega_{i}^{2}}}$
$\sigma_{y}$	$\sqrt{\frac{\sum_{i=1}^{n}{\left(\omega_{i}{\tilde{x}}_{i}\sin\theta -\omega_{i}{\tilde{y}}_{i}\cos\theta \right)}^{2}}{\sum_{i=1}^{n}\omega_{i}^{2}}}$

Where ($x_{i},y_{i}$) and ($\tilde{x_{i}},\tilde{y_{i}}$) denote the spatial location of the target area and the coordinate deviation from the target location to the mean center, respectively, while $w_{i}$ denotes the weight.

**Table 2:** Dagum’s Gini coefficient equations.

Index	Formula	Index	Formula
G	$\frac{\sum_{j=1}^{k}\sum_{h=1}^{k}\sum_{i=1}^{n_{j}}\sum_{r=1}^{n_{h}}\left|E_{ji}-E_{hr}\right|}{2n^{2}\bar{E}}$	$G_{w}$	$\sum_{j=1}^{k}G_{jj}p_{j}s_{j}$
$G_{jj}$	$\frac{\sum_{i=1}^{n_{j}}\sum_{r=1}^{n_{h}}\left|E_{ji}-E_{jr}\right|}{2n_{j}{}^{2}\bar{E_{j}}}$	$G_{nb}$	$\sum_{j=2}^{k}\sum_{h=1}^{j-1}G_{jh}\left(p_{j}s_{h}+p_{h}s_{j}\right)D_{jh}$
$G_{jh}$	$\frac{\sum_{i=1}^{n_{j}}\sum_{r=1}^{n_{h}}\left|E_{ji}-E_{jr}\right|}{n_{j}n_{h}\left(\bar{E_{j}}+\bar{E_{h}}\right)}$	$G_{t}$	$\sum_{j=2}^{k}\sum_{h=1}^{j-1}G_{jh}\left(p_{j}s_{h}+p_{h}s_{j}\right)\left(1-D_{jh}\right)$
$p_{j}=n_{j}/n$, $p_{k}=n_{k}/n$, $s_{j}=\frac{n_{j}\bar{E_{j}}}{n\bar{E}}$, $s_{h}=\frac{n_{h}\bar{E_{h}}}{n\bar{E}}$			

Where $E_{ji}(E_{hr})$ denotes the energy eco-efficiency of city i(r) in region j(h). The $\bar{E}$ and $\bar{E_{j}}(\bar{E_{h}})$ denote the mean value of energy eco-efficiency for the whole YRB and region j(h), respectively. The n and $n_{j}\left(n_{h}\right)$ denote the number of cities in the whole YRB and in region j(h), respectively. The k is the number of districts divided, and in this study its value is 3. The $D_{jh}$ denotes the relative impact of energy eco-efficiency between region j and region h.

**Table 3:** Input–output indicator system of energy eco-efficiency in the YRB.

Index	Variables	Indicator Explanation
Input	Capital	Capital stock of fixed assets
	Labor	Number of employees in secondary industry
	Energy	Comprehensive industrial energy consumption
Output	Economic	GDP of secondary industry
	Environment	Industrial SO_2_ emissions
		Industrial soot and sulfur emissions
		Industrial wastewater discharge
		CO_2_ emissions

**Table 4:** ML index and decomposition of energy eco-efficiency in the YRB from 2006–2018.

Year	ML	EC	TC
2006–2007	1.063	1.038	1.024
2007–2008	1.245	0.967	1.288
2008–2009	1.059	1.017	1.042
2009–2010	1.096	1.023	1.071
2010–2011	1.181	1.063	1.111
2011–2012	0.981	1.071	0.916
2012–2013	0.983	1.028	0.957
2013–2014	0.917	1.002	0.914
2014–2015	0.940	0.969	0.970
2015–2016	1.148	1.039	1.105
2016–2017	1.003	1.034	0.971
2017–2018	1.294	1.147	1.128
Mean	1.076	1.033	1.041
Upper	1.022	1.018	1.004
Middle	1.032	1.021	1.010
Lower	1.031	1.016	1.016

**Table 5:** Parameters of energy eco-efficiency SDEs in the YRB.

Year	Direction Angle/°	Standard Deviation Along *x*-Axis/km	Standard Deviation Along *y*-Axis/km
2006	91.636	6.181	2.673
2009	92.538	6.166	2.730
2012	91.858	6.248	2.782
2015	91.418	6.235	2.743
2018	91.934	6.111	2.696

**Table 6:** Dagum’s Gini coefficient and its decomposition results.

Year	Overall	Intra-Regional Difference			Inter-Regional Difference			Contribution Rates		
		Upper	Middle	Lower	Upper–Middle	Upper–Lower	Middle–Lower	*G_w_*	*G_nb_*	*G_t_*
2006	0.115	0.084	0.113	0.123	0.107	0.123	0.123	0.329	0.284	0.387
2007	0.114	0.104	0.099	0.121	0.108	0.130	0.118	0.322	0.256	0.422
2008	0.113	0.076	0.103	0.123	0.105	0.131	0.120	0.316	0.356	0.328
2009	0.112	0.098	0.100	0.116	0.107	0.129	0.115	0.320	0.270	0.410
2010	0.110	0.098	0.107	0.103	0.110	0.121	0.111	0.323	0.251	0.423
2011	0.106	0.095	0.100	0.102	0.112	0.119	0.102	0.322	0.245	0.432
2012	0.126	0.116	0.135	0.103	0.136	0.122	0.125	0.336	0.190	0.473
2013	0.128	0.093	0.129	0.123	0.135	0.133	0.130	0.325	0.267	0.408
2014	0.123	0.065	0.131	0.116	0.127	0.130	0.127	0.320	0.328	0.352
2015	0.126	0.065	0.136	0.118	0.122	0.135	0.134	0.320	0.339	0.341
2016	0.129	0.095	0.133	0.118	0.121	0.142	0.139	0.321	0.305	0.374
2017	0.146	0.140	0.151	0.138	0.147	0.143	0.147	0.341	0.103	0.556
2018	0.138	0.092	0.141	0.132	0.145	0.150	0.138	0.322	0.306	0.372

Note: *G_w_* refers to intra-regional differences; *G_nb_* refers to inter-regional differences; *G_t_* refers to the intensity of transvariation.

**Table 7:** Absolute β-convergence test results for the YRB during the period from 2006 to 2018.

Variable	Whole	Upper	Middle	Lower
β	−0.342 *** (0.031)	−0.458 *** (0.066)	−0.271 *** (0.046)	−0.379 *** (0.055)
_cons	0.262 *** (0.023)	0.298 *** (0.043)	0.215 *** (0.035)	0.3145 *** (0.045)
N	720	204	300	216
R-squared	0.154	0.207	0.141	0.193
Model	Fixed	Fixed	Fixed	Fixed
Convergence	Yes	Yes	Yes	Yes

Note: (1) The figures in parentheses below the parameter estimates are standard errors. (2) *** denotes rejection of the null hypothesis at the 1% level. (3) In accordance with the results of Hausman test, the fixed-effects regression model was selected.

**Table 8:** Conditional β -convergence test results for the YRB during the period from 2006 to 2018.

Variable	Whole	Upper	Middle	Lower
β	−0.418 *** (0.034)	−0.607 *** (0.071)	−0.325 *** (0.052)	−0.487 ** (0.061)
$LN\left(INN\right)$	0.016 ** (0.007)	0.017 (0.011)	0.012 (0.013)	0.048 ** (0.019)
OPE	−0.286 * (0.256)	−0.323 * (0.174)	−0.957 * (0.510)	−0.191 (0.429)
GOV	−0.023 (0.080)	0.030 (0.096)	−0.058 (0.185)	0.541 (0.511)
URB	0.002 ** (0.001)	0.002 * (0.001)	0.002 (0.003)	−0.004 (0.003)
STR	−0.079 *** (0.017)	−0.119 *** (0.027)	−0.050 * (0.031)	−0.006 (0.046)
_cons	0.218 *** (0.034)	0.351 *** (0.064)	0.169 ** (0.069)	0.242 *** (0.072)
N	720	204	300	216
R-squared	0.197	0.311	0.147	0.260
Model	Fixed	Fixed	Fixed	Fixed
Convergence	Yes	Yes	Yes	Yes

Note: (1) The figures in parentheses below the parameter estimates are standard errors. (2) ***, **, and * denote rejection of the null hypothesis at the 1%, 5%, and 10% levels, respectively. (3) In accordance with the results of Hausman test, the fixed-effects regression model was selected.

## Data Availability

The data that support the findings of this study are openly available in CNKI at https://kns.cnki.net/kns8?dbcode=CYFD, accessed on 7 November 2022.
